# Chronic stress and corticosterone exacerbate alcohol-induced tissue injury in the gut-liver-brain axis

**DOI:** 10.1038/s41598-020-80637-y

**Published:** 2021-01-12

**Authors:** Pradeep K. Shukla, Avtar S. Meena, Kesha Dalal, Cherie Canelas, Geetha Samak, Joseph F. Pierre, RadhaKrishna Rao

**Affiliations:** 1 Department of Physiology, College of Medicine, University of Tennessee Health Science Center, 3 N Dunlap, Suite S303, Memphis, TN 38163 USA; 2 Department of Pediatrics, College of Medicine, University of Tennessee Health Science Center, Memphis, TN 38163 USA

**Keywords:** Colon, Gastrointestinal models

## Abstract

Alcohol use disorders are associated with altered stress responses, but the impact of stress or stress hormones on alcohol-associated tissue injury remain unknown. We evaluated the effects of chronic restraint stress on alcohol-induced gut barrier dysfunction and liver damage in mice. To determine whether corticosterone is the stress hormone associated with the stress-induced effects, we evaluated the effect of chronic corticosterone treatment on alcoholic tissue injury at the Gut-Liver-Brain (GLB) axis. Chronic restraint stress synergized alcohol-induced epithelial tight junction disruption and mucosal barrier dysfunction in the mouse intestine. These effects of stress on the gut were reproduced by corticosterone treatment. Corticosterone synergized alcohol-induced expression of inflammatory cytokines and chemokines in the colonic mucosa, and it potentiated the alcohol-induced endotoxemia and systemic inflammation. Corticosterone also potentiated alcohol-induced liver damage and neuroinflammation. Metagenomic analyses of 16S RNA from fecal samples indicated that corticosterone modulates alcohol-induced changes in the diversity and abundance of gut microbiota. In Caco-2 cell monolayers, corticosterone dose-dependently potentiated ethanol and acetaldehyde-induced tight junction disruption and barrier dysfunction. These data indicate that chronic stress and corticosterone exacerbate alcohol-induced mucosal barrier dysfunction, endotoxemia, and systemic alcohol responses. Corticosterone-mediated promotion of alcohol-induced intestinal epithelial barrier dysfunction and modulation of gut microbiota may play a crucial role in the mechanism of stress-induced promotion of alcohol-associated tissue injury at the GLB axis.

## Introduction

Alcohol-related diseases (ARD) are the primary health issues across populations worldwide. A common denominator in the pathogenesis of ARD is systemic inflammation^1, 2^. Alcohol induces inflammatory responses in multiple organs, including the gut, liver, pancreas, and brain^1^. Gut barrier dysfunction and resulting endotoxemia are crucial steps involved in systemic inflammation in alcohol use disorders (AUD)^3^. Intestinal dysbiosis, associated with increased production of bacterial toxins, is another essential cofactor in alcohol-associated endotoxemia. The intestinal microbial lipopolysaccharide (LPS) is absorbed into the portal circulation and targets the Kupffer cells and hepatocytes in the liver. The LPS-activated liver cells release a variety of pro-inflammatory cytokines and chemokines^4–6^. Once released into the systemic circulation, LPS targets multiple organs by inducing a systemic inflammatory response. For instance, alcohol induces neuroinflammation potentially by LPS and toll-like receptor 4-dependent mechanism^7–10^.

The primary component of the intestinal mucosal barrier function is the epithelial tight junction (TJ), which prevents diffusion of LPS from the gut lumen into the mucosa and subsequent absorption into the systemic circulation. The TJ is a multiprotein complex consisting of various proteins, including occludin, claudins, zonula occludens (ZO-1), and cingulin^11–13^. The TJ protein complex is fastened to the actomyosin belt at the apical end of the epithelial cells. Ethanol and its metabolite, acetaldehyde, synergistically disrupt the intestinal epithelial TJ by involving multiple intracellular signaling mechanisms^14–16^. TJ disruption is a crucial step involved in alcohol-induced endotoxemia. The adherens junction (AJ) consists of E-cadherin, β-catenin, and other proteins^17^. It is located beneath the TJ and indirectly regulates the integrity of TJ^18^.

Only about 20% of alcoholics develop liver disease^19^. Therefore, the multiple-hit-model is proposed to describe the pathogenesis of alcohol-associated liver disease^20^. Chronic stress plays an essential role in the pathogenesis of many gastrointestinal and systemic diseases^21–24^. AUD is highly comorbid with chronic stress disorders^25–27^. The stress-alcohol interaction studies, so far, have been focused on alcohol use and dependence. Evidence indicates that stress is a trigger for relapse of alcohol use and motivation to drink^28^. However, specific interactions between the alcohol and stress circuitries are unclear^25^. A crucial pathway altered during alcohol use and dependence is the Hypothalamic–Pituitary–Adrenal (HPA) axis^29^. Acute alcohol intake causes a stress-like response that includes a transient increase in plasma cortisol^30^. In contrast, chronic alcohol use leads to dysregulation of the HPA axis and a sustained elevation of plasma cortisol^30^. Cortisol causes multiple cellular effects in different tissues by binding to a ubiquitously distributed intracellular glucocorticoid receptor (GR)^31^. Many studies have addressed the role of stress, glucocorticoids, and glucocorticoid receptors in alcohol use and dependence. However, there is no information available regarding the impact of stress or glucocorticoids on alcohol-induced tissue injury. We hypothesize that chronic stress is a potential a secondary hit in the pathogenesis of alcohol-related diseases, exacerbating alcohol-induced tissue damage. In the current study, we investigated the cross-talk between chronic stress (or corticosterone) and alcohol, leading to synergistic intestinal epithelial permeability, dysbiosis, and systemic responses.

## Results

### Restraint stress exacerbates alcohol-induced mucosal barrier dysfunction in the mouse intestine

**Figure 1:**
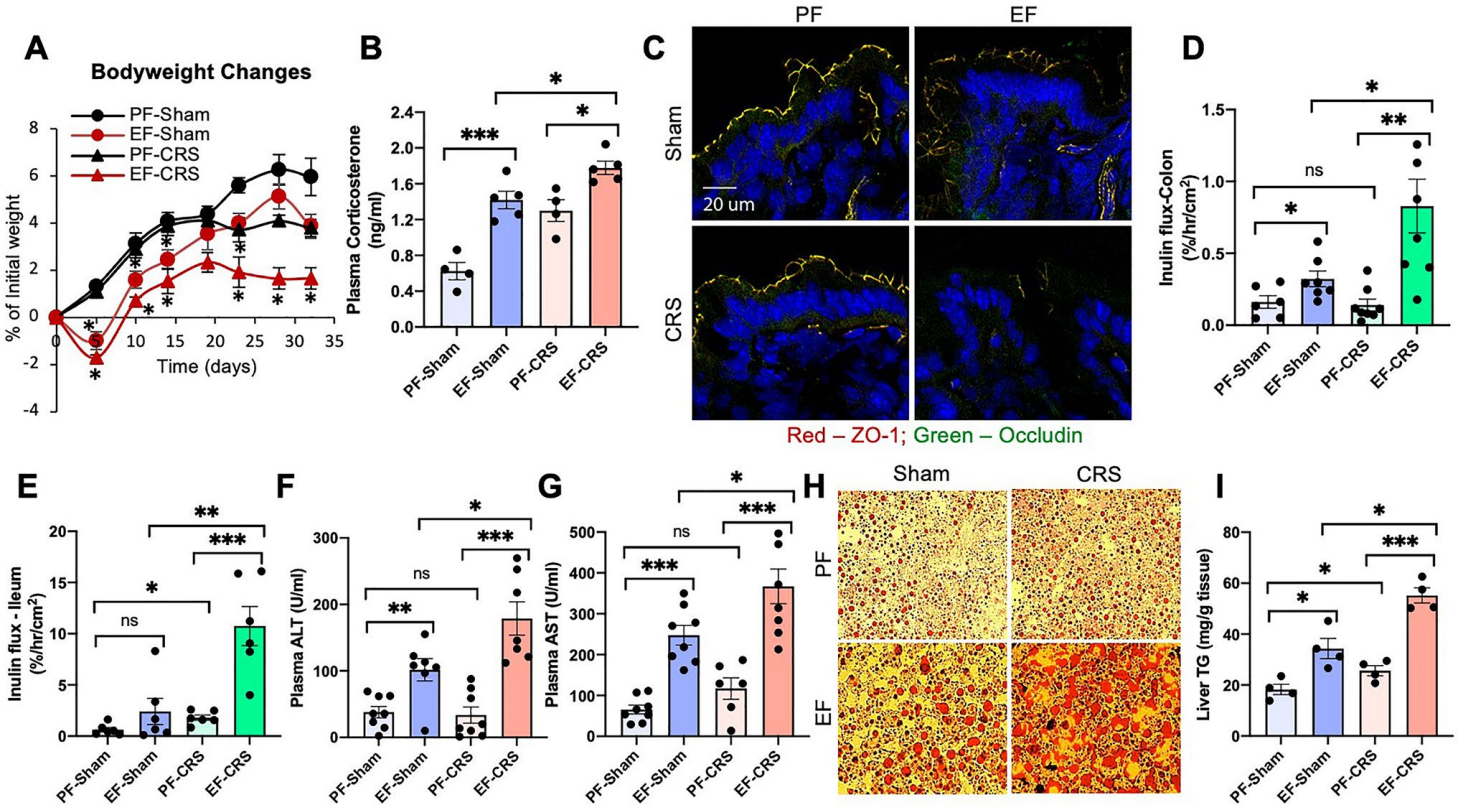
Chronic restraint stress synergizes alcohol-induced disruption of gut barrier dysfunction and liver damage. Adult mice were fed a liquid diet with (EF) or without (PF) ethanol for four weeks. In some groups, animals were subjected to two-hour restraint stress (CRS) or "Sham" treated. (**A**) Body weights were recorded twice a week. (**B**) Plasma corticosterone levels. (**C**) Epithelial tight junction integrity was assessed by staining cryosections of the colon for occludin and ZO-1 by immunofluorescence method followed by confocal imaging. (**D**,**E**) Mucosal permeability was measured by the vascular-to-luminal flux of FITC-inulin in the colon (**D**) and ileum (**E**) in vivo. (**F**,**G)** ALT (**F**) and AST (**G**) activities were measured in plasma. (**H**,**I**) Liver sections were stained with Oil-Red-O for fat deposits (**H**). Liver extracts were analyzed for triglyceride content (**I**). Values in graphs are mean ± SEM (n = 6). Dots in bars indicate individual values. The numbers above the bars are *p*-values for differences between the groups indicated by the horizontal lines, and "ns" indicates no significant difference between groups.

As described above, chronic stress is likely a second-hit in the pathogenesis of alcohol-associated tissue injury. Mice were subjected to chronic ethanol feeding or chronic restraint stress (CRS) alone and ethanol feeding combined with CRS (EtOH + CRS). The diet intake in different groups was matched by pair-feeding. CRS initially caused a significant loss of body weight in pair-fed and ethanol-fed mice, which recovered subsequently (Fig. 1A). Body weights in the EtOH + CRS group, however, remained significantly lower than in the other groups through the course of the experiment. Plasma corticosterone levels were significantly increased by ethanol feeding, and the levels several-fold higher when ethanol feeding was combined with CRS (Fig. 1B). The corticosterone levels were measured at the end of the experiment, and therefore it represents the steady-state concentrations. The plasma corticosterone concentration is likely much higher during restraint stress. Confocal microscopic images show that ethanol and CRS by themselves caused a redistribution of occludin and ZO-1 from the intercellular junctions of the colonic epithelium (Fig. 1C); these effects were more severe in the EtOH + CRS group. Redistribution of TJ proteins by ethanol and CRS was associated with a significant increase in mucosal inulin permeability in the colon (Fig. 1D). The increase in colonic mucosal permeability in the EtOH + CRS group was higher than the sum of ethanol and CRS effects alone. In the ileum, ethanol and CRS alone showed no significant alteration of mucosal permeability (Fig. 1E). However, EtOH + CRS increased inulin permeability by nearly four folds. These data indicate that CRS promotes alcohol-induced gut barrier dysfunction.

### CRS enhances alcohol-induced liver damage

Chronic alcohol use causes fatty liver and hepatitis^32^. We examined the potential influence of chronic stress on alcohol-induced liver injury. Ethanol feeding significantly elevated plasma alanine transaminase (ALT; Fig. 1F) and aspartate transaminase (AST; Fig. 1G). CRS alone caused no effect on plasma levels of ALT or AST, but it significantly enhanced the ethanol-induced increase in these liver enzymes. Images from Oil Red O staining of liver sections showed that ethanol increased lipid deposits in the liver (Fig. 1H), associated with a significant increase in liver triglyceride content (Fig. 1I). CRS caused no significant effect on liver triglycerides, but it potentiated the effects of ethanol on lipid deposition and triglyceride content. Data indicate that CRS synergizes the damaging effects of alcohol on the liver.

### Corticosterone promotes alcohol-induced TJ disruption and mucosal barrier dysfunction in mouse colon in vivo

**Figure 2:**
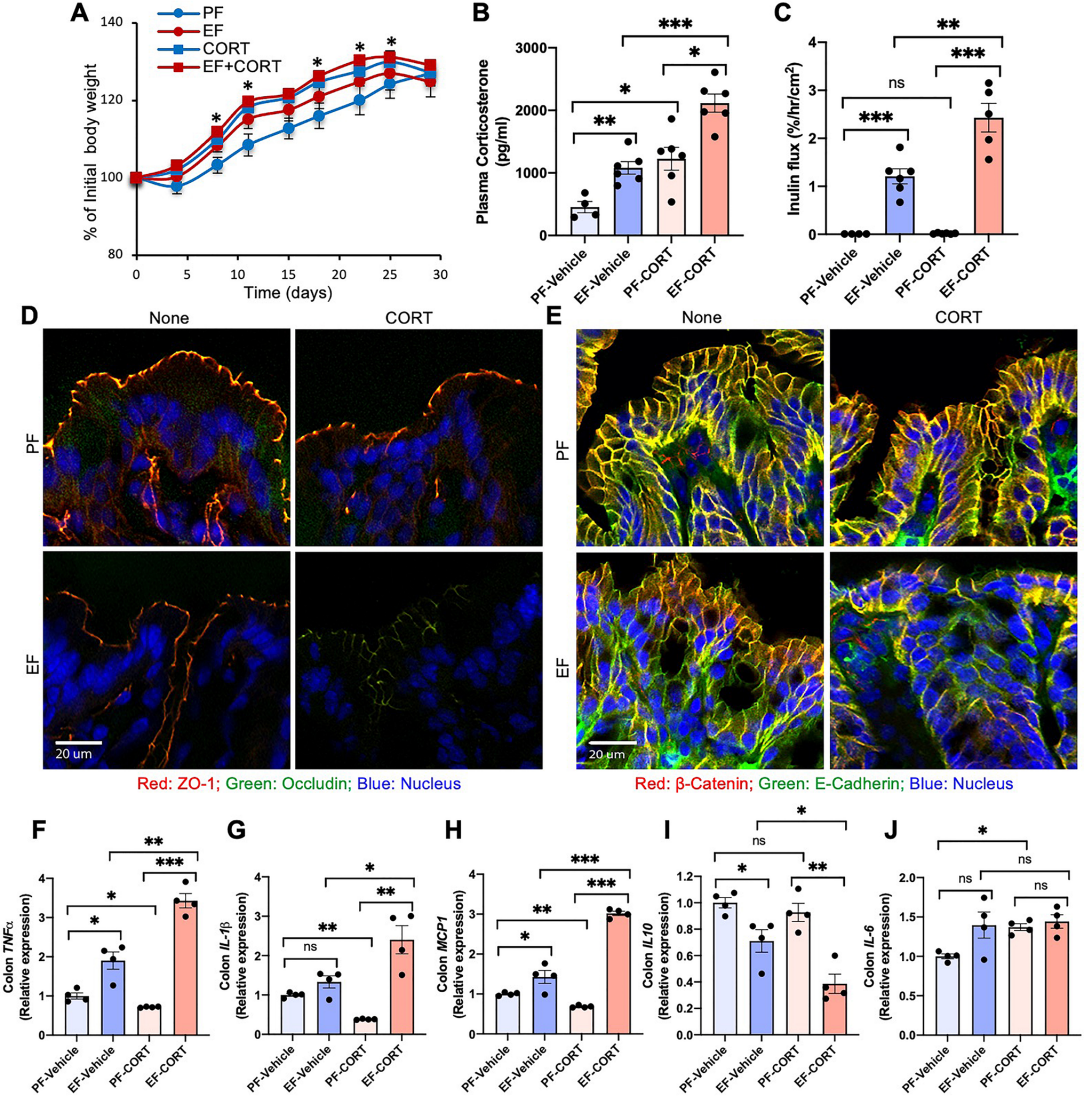
Corticosterone enhances alcohol-induced barrier dysfunction and mucosal inflammatory responses in the mouse colon. Adult mice were fed a liquid diet with (EF) or without (PF) ethanol for four weeks. In some groups, animals were injected with corticosterone (CORT) daily. Animals in other groups were injected with the vehicle. (**A**) Body weights were recorded twice a week. (**B**) Corticosterone levels were measured in plasma. (**C**) Mucosal permeability was measured by the vascular-to-luminal flux of FITC-inulin in the colon in vivo. (**D**,**E**) Epithelial junctional integrity was assessed by staining the cryosections of the colon for occludin and ZO-1 for tight junction (**D**) and E-cadherin and β-catenin for adherens junction (**E**) by immunofluorescence method followed by confocal imaging. (**F–J**) RNA isolated from colonic mucosa was analyzed for mRNA specific for *TNFα* (**D**), *IL-1β* (**E**), *MCP1* (**F**), and *IL-10* (**G**) by RT-qPCR. Values in graphs are mean ± SEM (n = 4–6 for **A**–**C** and 4 for **F**–**J**). Dots in bars indicate individual values. The numbers above the bars are *p*-values for differences between the groups indicated by the horizontal lines, and "ns" indicates no significant difference between groups.

The CRS effect could be mediated by several factors, such as corticosterone and epinephrine. As corticosterone was previously shown to cause intestinal barrier dysfunction, we wanted to determine whether the stress-induced effect was mediated by corticosterone. Hence, the second study was performed to determine whether chronic corticosterone treatment influenced ethanol-induced tissue injury. Bodyweight gains were significantly higher in mice that received ethanol and corticosterone (Fig. 2A), even though the diet intake was maintained similarly in all groups by pair-feeding. Chronic ethanol feeding and corticosterone treatment both elevated plasma corticosterone more than two-fold (Fig. 2B). Plasma corticosterone was measured 24 h after the last injection of corticosterone. Therefore, it is the steady-state concentration of corticosterone. This dose of corticosterone was previously reported to show stress responses^33^. This steady-state concentration of corticosterone is similar to the corticosterone concentration recorded after CRS (Fig. 1B). The combination of ethanol and corticosterone (EtOH + CORT) caused a four-fold increase in plasma corticosterone. Ethanol, corticosterone, or EtOH + CORT caused no visible morphologic changes in the colonic mucosa (Fig. ). Corticosterone alone did not affect colonic mucosal permeability to inulin, but it significantly potentiated ethanol-induced mucosal permeability (Fig. 2C). The confocal microscopic images showed that corticosterone enhanced the ethanol-induced redistribution of TJ (Fig. 2D) and AJ (Fig. 2E) proteins from the colonic epithelial junctions. These results indicate that corticosterone potentiates alcohol-induced disruption of colonic epithelial barrier function.

### Corticosterone potentiates ethanol-induced inflammatory response in the colonic mucosa

**Figure 3:**
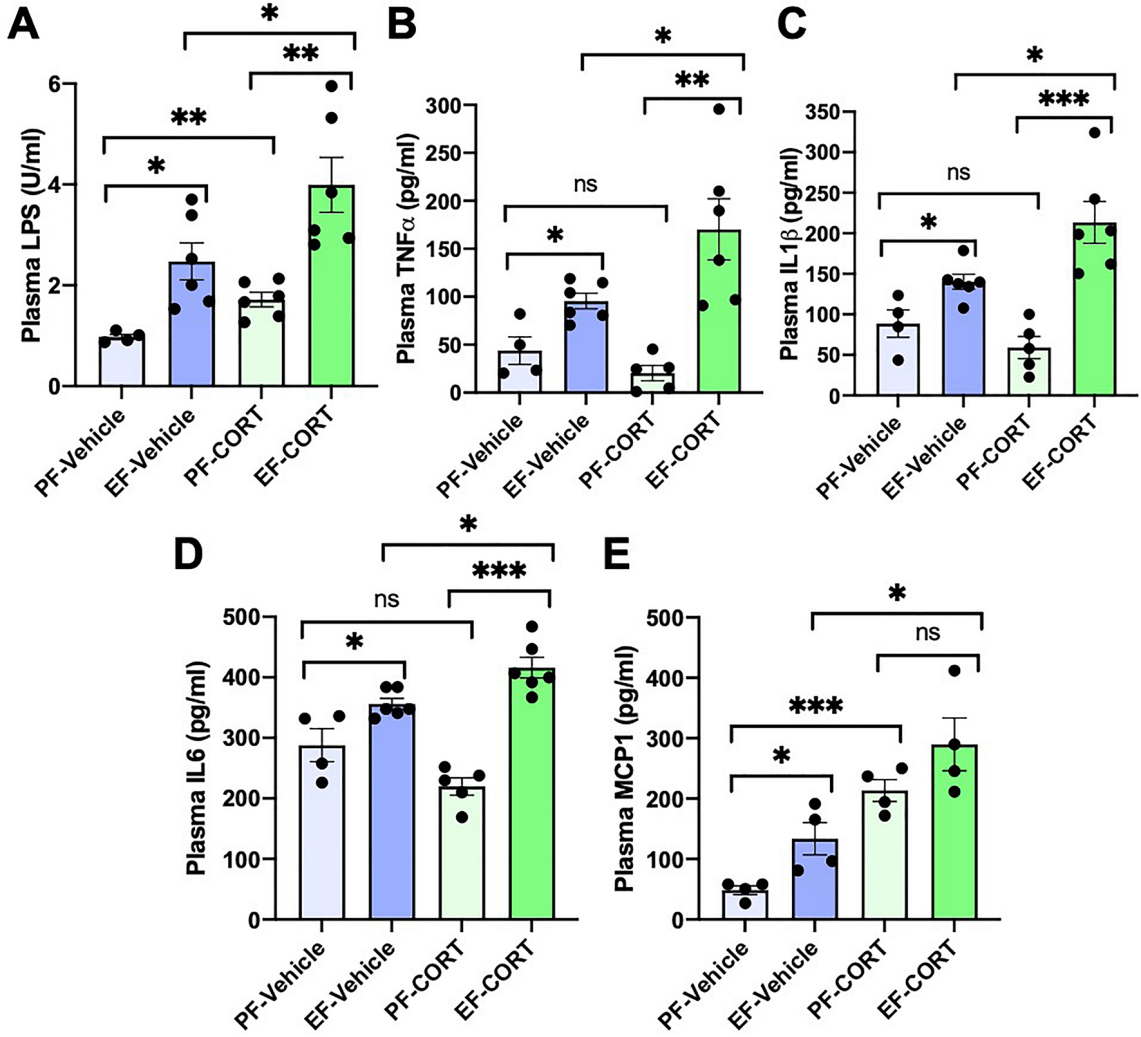
Corticosterone exacerbates alcohol-induced endotoxemia and systemic inflammation. Adult mice were fed a liquid diet with (EF) or without (PF) ethanol for four weeks. In some groups, animals were injected with corticosterone (CORT) daily. Animals in other groups were injected with the vehicle. (**A**) Endotoxemia was assessed by measuring plasma LPS levels. (**B**–**D**) Levels of TNFα (**B**), IL-1β (**C**), IL-6 (**D**), and MCP1 (**E**) were measured in plasma by ELISA. Values in graphs are mean ± SEM (n = 6, except 4 for the PF-Vehicle group). Dots in bars indicate individual values. The numbers above the bars are *p*-values for differences between the groups indicated by the horizontal lines, and "ns" indicates no significant difference between groups.

Disruption of intestinal epithelial TJ opens the gateway for the influx of bacterial toxins (LPS) from the intestinal lumen into the mucosa, where it stimulates epithelial and immune cells and triggers an inflammatory response. We measured mRNA for different cytokines and the chemokine monocyte chemoattractant protein 1 (MCP1) as the indicator for inflammatory responses in the colon. Ethanol increased mRNA for tumor necrosis factor-α (*TNFα*; Fig. 3F), interleukin-1β (*IL-1β*; Fig. 2G), and *MCP1* (Fig. 2H). Corticosterone by itself caused a significant reduction of *IL-1β*, *TNFα*, and *MCP1* mRNA levels; however, it potentiated ethanol-induced elevation of mRNA for these cytokines and chemokine. Ethanol feeding significantly reduced interleukin-10 (*IL-10*) mRNA levels (Fig. 2I). Corticosterone did not alter *IL-10* mRNA, but it potentiated the effect of ethanol. Ethanol or corticosterone alone or together had no significant effect on *IL-6* mRNA in the colon (Fig. 2J). These data indicate that corticosterone synergizes alcohol-induced inflammatory responses in the mouse colon.

### Exacerbation of alcohol-induced systemic inflammation by corticosterone

A consequence of gut barrier disruption is the absorption of LPS into the portal circulation and subsequent delivery into the systemic circulation, a condition referred to as endotoxemia. The current study shows that ethanol and corticosterone caused a significant elevation of plasma LPS (Fig. 3A). However, EtOH + CORT-induced elevation of plasma LPS levels was synergistically higher than by ethanol or corticosterone alone. Endotoxemia is known to induce inflammatory responses in various organs. Therefore, we measured levels of representative cytokines in plasma as a gauge of systemic inflammation. Ethanol significantly elevated plasma TNFα (Fig. 3B), IL-1β (Fig. 3C), IL-6 (Fig. 3D), and MCP1 (Fig. 3E). Corticosterone by itself caused a significant reduction of plasma TNFα, IL-1β, and IL-6; however, it potentiated the ethanol-induced elevation of plasma cytokines. Corticosterone elevated plasma MCP1, but the levels were significantly higher by EtOH + CORT than in EtOH or corticosterone alone. These data indicate that corticosterone enhances alcohol-induced endotoxemia and systemic inflammation.

### Corticosterone reinforces alcohol-induced liver damage

**Figure 4:**
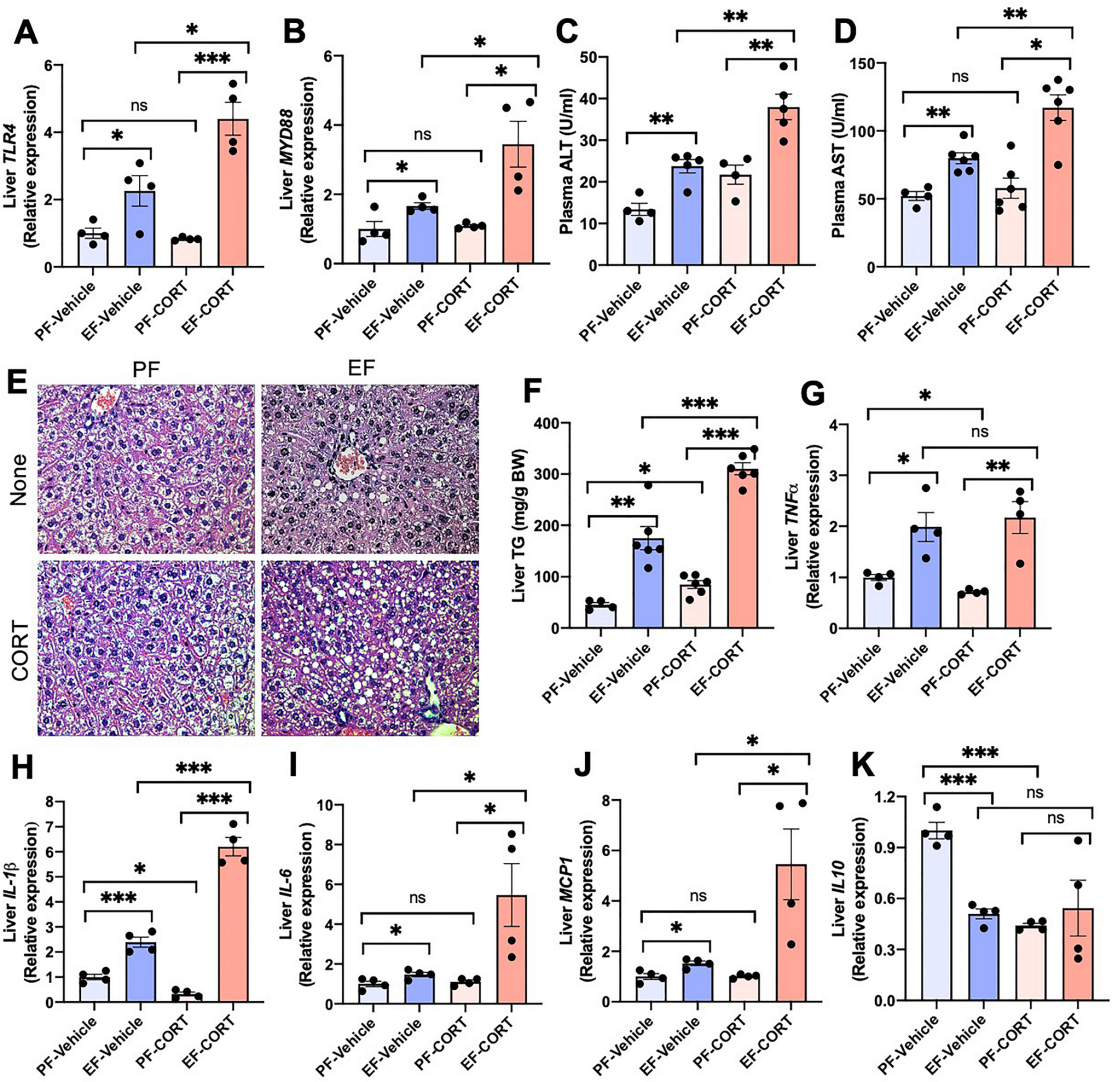
Corticosterone potentiates alcohol-induced liver damage. Adult mice were fed a liquid diet with (EF) or without (PF) ethanol for four weeks. In some groups, animals were injected with corticosterone (CORT) daily. Animals in other groups were injected with the vehicle. (**A**,**B**) Expression of *TLR4* (**A**) and *MYD88* (**B**) in the liver was assessed by RT-qPCR for specific mRNA. (**C**,**D**) Plasma was analyzed for ALT (**C**) and AST (**D**) activities. (**E**) Liver histopathology was performed by H&E staining and bright field microscopy. (**F**) Steatosis was assessed by measuring liver triglyceride content. (**G–K**) Inflammatory responses in the liver were determined by measuring specific mRNA for *TNFα* (**G**), *IL-1β* (**H**), *IL-6* (**I**), *MCP1* (**J**), and *IL-10* (**K**). Values in graphs are mean ± SEM (n = 6 for **A**–**E**, and 4 for **F**–**J**). Dots in bars indicate individual values. The numbers above the bars are *p*-values for differences between the groups indicated by the horizontal lines, and "ns" indicates no significant difference between groups.

Evidence suggests that gut barrier dysfunction and endotoxemia are causal factors in the mechanism of alcohol-associated liver damage. The liver is the first target of portal LPS. LPS exposure has been shown to increase the expression of TLR4 and downstream signaling elements in a variety of tissues^34,35^. We measured the levels of mRNA for *TLR4* and *MYD88* in the liver as a marker of LPS exposure. Ethanol feeding significantly elevated both *TLR4* (Fig. 4A) and myeloid differentiation primary response-88 (*MYD88*; Fig. 4B) mRNA in the liver. Corticosterone alone did not affect *TLR4* or *MYD88* mRNA; however, it enhanced the ethanol-induced elevation of *TLR4* and *MYD88* mRNA by several folds. Ethanol, but not corticosterone, elevated plasma ALT (Fig. 4C) and AST (Fig. 4D). Corticosterone enhanced the ethanol effect on AST by several folds. Histopathologic analysis indicated that corticosterone increased the severity of ethanol-induced liver injury (Fig. 4E). Corticosterone also enhanced the ethanol-induced elevation of liver triglycerides (Fig. 4F). Data indicate that corticosterone promotes alcohol-associated liver damage.

We assessed the inflammatory responses in the liver by measuring *TNFα*, *IL-1β*, *IL-6*, and *MCP1* mRNA levels. Ethanol feeding significantly increased liver mRNA for *TNFα* (Fig. 4G), *IL-1β* (Fig. 4H), interleukin-6 (*IL-6*; Fig. 4I), and *MCP1* (Fig. 4J). Corticosterone by itself caused a significant reduction of *TNFα* and *IL-1β* mRNA in the liver. However, corticosterone elevated ethanol-induced increase in liver mRNA for *IL-1β*, *IL-6*, and *MCP1* by 4–12 fold. Ethanol, corticosterone, and EtOH + CORT caused a similar reduction of *IL-10* mRNA in the liver (Fig. 4K). These results indicate that alcohol-induced inflammatory reactions in the liver are enhanced by corticosterone.

### Corticosterone potentiates alcohol-induced neuroinflammation

**Figure 5:**
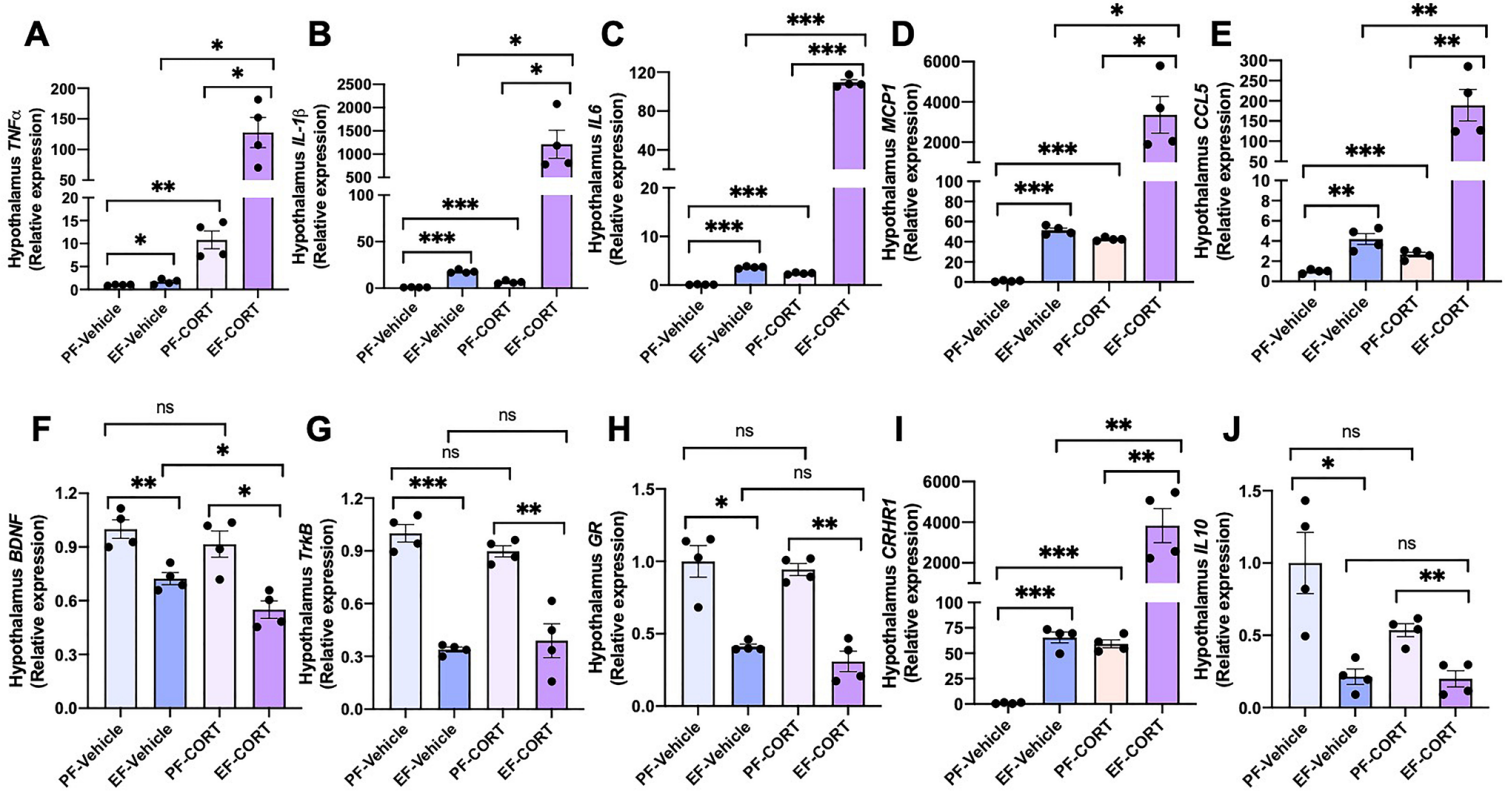
Corticosterone synergizes alcohol-induced neuroinflammation. Adult mice were fed a liquid diet with (EF) or without (PF) ethanol for four weeks. In some groups, animals were injected with corticosterone (CORT) daily. Animals in other groups were injected with the vehicle. Inflammatory responses in the hypothalamus were assessed by measuring specific mRNA for *TNFα* (**A**), *IL-1β* (**B**), *IL-6* (**C**), *MCP1* (**D**), *CCL5* (**E**), *BDNF* (**F**), *TrkB* (**G**), *GR* (**H**), *CRHR1* (**I**), and *IL-10* (**J**). Values in graphs are mean ± SEM (n = 4). Dots in bars indicate individual values. The numbers above the bars are *p*-values for differences between the groups indicated by the horizontal lines, and "ns" indicates no significant difference between groups.

Neuroinflammation and dysregulated HPA axis are implicated in various ARD phenotypes. However, no information is available regarding the impact of the altered HPA axis in alcohol-associated tissue injury. Glucocorticoids have been established as a suppressor of neuroinflammation^36,37^. However, chronic glucocorticoid treatment is suggested to promote neuroinflammation^38,39^. We examined the effect of alcohol and corticosterone on cytokine and chemokine expression in the hypothalamus. The data showed that ethanol significantly elevates mRNA for *TNFα* (Fig. 5A), *IL-1β* (Fig. 5B), *IL-6* (Fig. 5C), *MCP1* (Fig. 5D), and chemokine ligand-5 (*CCL5*; Fig. 5E) in the hypothalamus. Ethanol feeding elevated mRNA for these cytokines and chemokines by 4–50 fold, and corticosterone alone raised mRNA for these cytokines and chemokines by 2–24 fold. Corticosterone induced a robust potentiation of ethanol-induced increase in mRNA for these cytokines and chemokines by 130–4000 fold over basal levels. Brain-derived neurotrophic factor (BDNF) and its receptor, tropomyosin receptor kinase B (TrkB), show anti-inflammatory influences in the brain^40,41^. Our data show that ethanol feeding significantly reduced *BDNF* (Fig. 5F) and *TrkB* (Fig. 5G) mRNA levels in the hypothalamus. Corticosterone did not influence ethanol's effects on *TrkB* mRNA, but it significantly potentiated ethanol's effect on *BDNF* mRNA. The mRNA for GR was reduced by ethanol feeding (Fig. 5H), but corticosterone did not affect either in the presence or absence of ethanol. On the other hand, corticosterone and ethanol demonstrated a robust synergy in elevating mRNA for the corticotropin-releasing hormone receptor-1 (*CRHR1*) gene (Fig. 5I). These data indicate a robust synergy between ethanol and corticosterone in inducing inflammatory responses in the hypothalamus. Ethanol and corticosterone significantly reduced *IL-10* mRNA, but corticosterone did not influence ethanol-induced down-regulation of *IL-10* expression (Fig. 5J).

### Corticosterone sensitizes Caco-2 cell monolayers for ethanol and acetaldehyde-induced TJ disruption and barrier dysfunction

**Figure 6:**
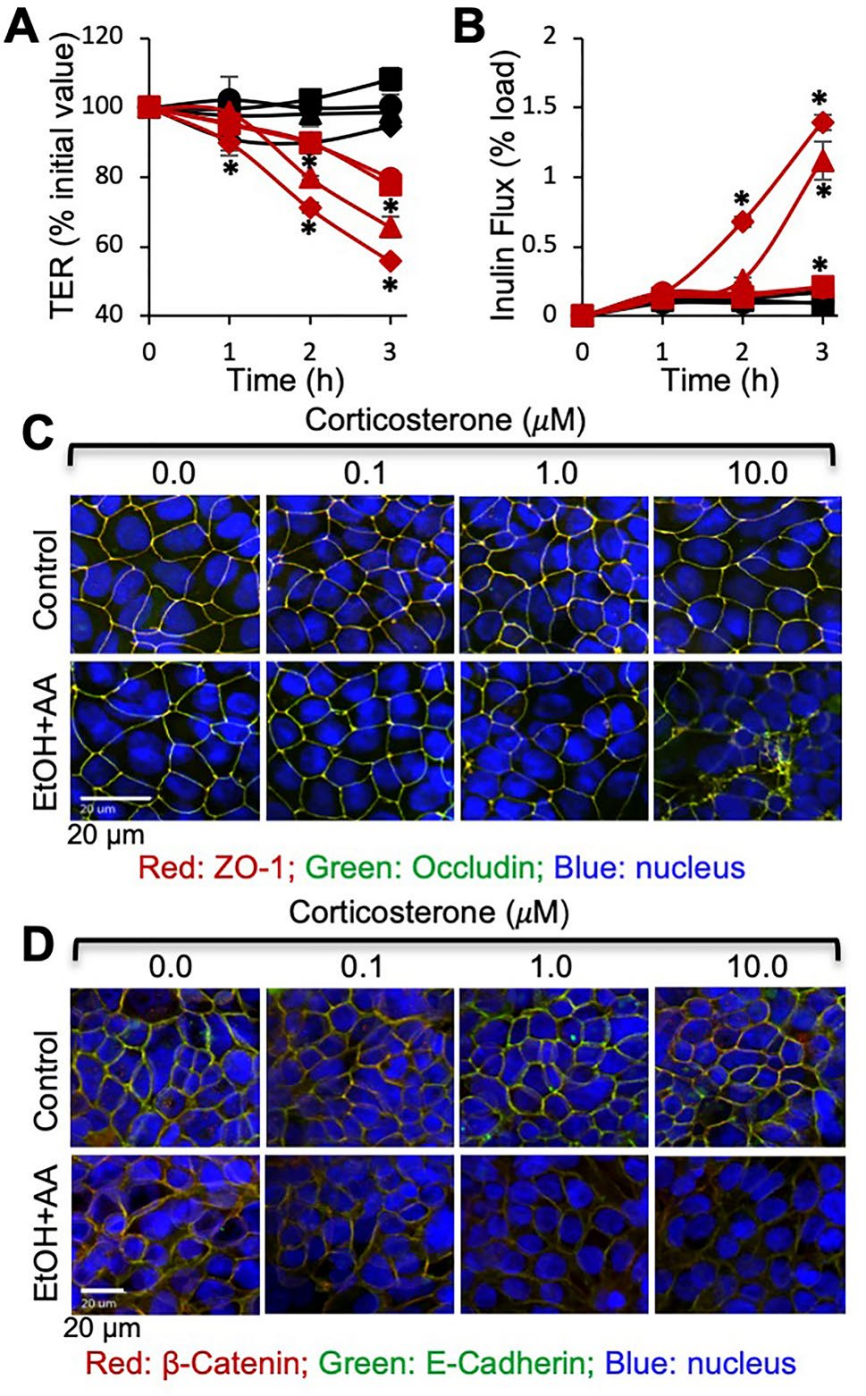
Corticosterone sensitizes Caco-2 cell monolayers for ethanol and acetaldehyde-induced tight junction disruption and barrier dysfunction. Caco-2 cell monolayers were pretreated with varying concentrations (black circle, red circle 0 μM, black square, red square 0.1 μM, black triangle, red triangle 1.0 μM, black diamond, red diamond 10 μM) of corticosterone (CORT) for 24 h, followed by incubation with (red circle, red square, red triangle, red diamond) or without (black circle, black square, black triangle, black diamond) ethanol (20 mM) and acetaldehyde (100 μM) (EtOH + AA). At varying times, TER (**A**) and FITC-inulin permeability (**B**) were measured. At 3 h of incubation, cell monolayers were fixed and co-stained for occludin and ZO-1 (**C**) or E-cadherin and β-catenin (**D**). Values in graphs are mean ± SEM (n = 6). Asterisks indicate the values that are significantly (P < 0.05) different from corresponding values for groups without ethanol and acetaldehyde treatments.

The primary mechanism involved in endotoxemia is the disruption of intestinal epithelial tight junction, epithelial barrier dysfunction, and LPS absorption. In the in vivo studies, it is unclear whether corticosterone directly affects the intestinal epithelium to increase gut permeability or if it was an indirect effect via corticosterone effect on other tissues. To determine the direct impact of corticosterone on the epithelium, we examined the effect of corticosterone (24-h pretreatment) on the ethanol and acetaldehyde (EtOH + AA)-induced TJ disruption and barrier dysfunction in Caco-2 cell monolayers. Our previous study demonstrated that ethanol and acetaldehyde synergistically disrupt TJ in Caco-2 cell monolayers^16^. In the current study, we used sub-responsive ethanol and acetaldehyde concentrations to evaluate the synergistic effects of corticosterone. Under these conditions, corticosterone or EtOH + AA produced no significant effect on the transepithelial electrical resistance (TER) (Fig. 6A) or inulin permeability in sham-treated cell monolayers (Fig. 6B). However, EtOH + AA decreased TER and increased inulin permeability in cell monolayers pre-treated with corticosterone. The confocal microscopic images show that the junctional distribution of TJ and AJ proteins in Caco-2 cell monolayers were unaffected by corticosterone or EtOH + AA. Nevertheless, the corticosterone and EtOH + AA combination induced a robust redistribution of occludin and ZO-1 (Fig. 6C) as well as E-cadherin and β-catenin (Fig. 6D) from the intercellular junctions. The magnitude of these synergistic effects of corticosterone on EtOH + AA-induced junctional disruption and barrier dysfunction was dose-dependent.

### Corticosterone modulates alcohol-induced intestinal dysbiosis

**Figure 7:**
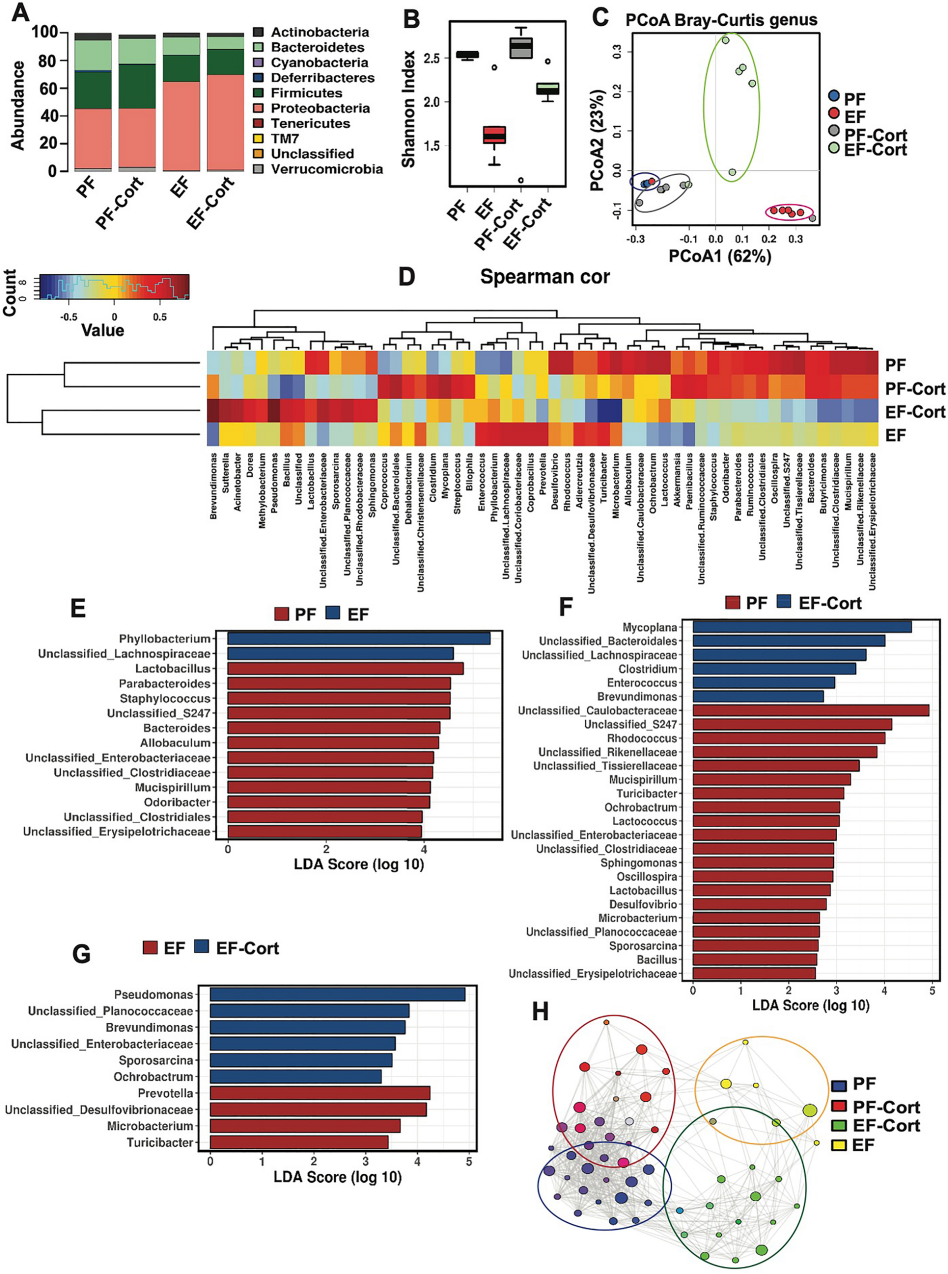
Corticosterone effect alcohol-induced intestinal dysbiosis: taxonomic analysis. Adult mice were fed a liquid diet with (EF) or without (PF) ethanol for four weeks. In some groups, animals were injected with corticosterone (CORT) daily. Animals in other groups were injected with the vehicle. Analysis of data from 16S rRNA-sequencing of fecal samples from different groups of mice is presented. (**A**) Relative abundance of different phyla of bacteria. (**B**) The Shannon Index was used to quantify α-diversity. (**C**) Principal coordinate analysis (PCoA) based on Bray–Curtis dissimilarity analysis was performed to determine β-diversity. (**D**) Spearman's correlation of microbiota at the genus level in different experimental groups. The dendrogram illustrates the genus and experimental group clustering**. **(**E**) Linear discriminate analysis of effect size (LefSe) was used to identify enriched taxa following ethanol feeding and corticosterone administration. (**F**) The network analysis of genus clustering was calculated by Spearman's correlations, where nodes represent genera associated and the experimental groups coded by color.

The second primary mechanism involved in endotoxemia is the dysbiosis of intestinal microbiota, leading to an increase in the relative abundance of pathobionts and LPS production. Previous studies by metagenomic analyses demonstrated that alcohol consumption leads to significant alteration of the gut microbiota composition^42,43^. We performed 16S ribosomal RNA (rRNA) gene sequencing and taxonomic analyses of fecal microbiota to determine whether corticosterone modified ethanol-induced dysbiosis. Alcohol feeding altered the taxonomic composition of fecal microbiota (Fig. 7A). Evaluation of α-diversity by analyzing the Shannon indices at the genus level indicated that ethanol caused a robust decrease in the Shannon index, whereas corticosterone significantly blocked the ethanol-induced reduction in the Shannon index (Fig. 7B). Beta-diversity analysis by Bray–Curtis indicated significant microbial composition differences between different groups (Fig. 7C). Spearman's correlation analysis, displayed as a heat map, confirmed substantial differences among the groups at the genus level (Fig. 7D). Spearman's correlation and linear discriminant analysis effect size (LEfSe) indicated that ethanol feeding selectively altered numerous genera of bacteria (Fig. 7E); these alterations of gut microbiota were much severe when ethanol feeding was combined with corticosterone treatment (Fig. 7F). The effects of ethanol and corticosterone on the abundance of selected phyla are presented in the Supplemental Information (Fig. S2). Interestingly, corticosterone caused a robust increase in the relative abundance of *Enterobacteriaceae, Pseudomonas*, and *Brevundimonas* in ethanol-fed mice (Fig. 7G).

We performed network analysis by Spearman’s correlation, which displays taxa likely to co-occur, color-coded by respective experimental groups (Fig. 7H) where node size indicates relative importance, and the lines indicate positive co-associations. Consistent with our other computational approaches, the network data illustrate distinct community membership changes following ethanol feeding. The network data demonstrated that the ethanol-induced changes occurred in clusters, suggesting that community stability or function may be globally altered after ethanol feeding; corticosterone further modulated ethanol-induced changes.

**Figure 8:**
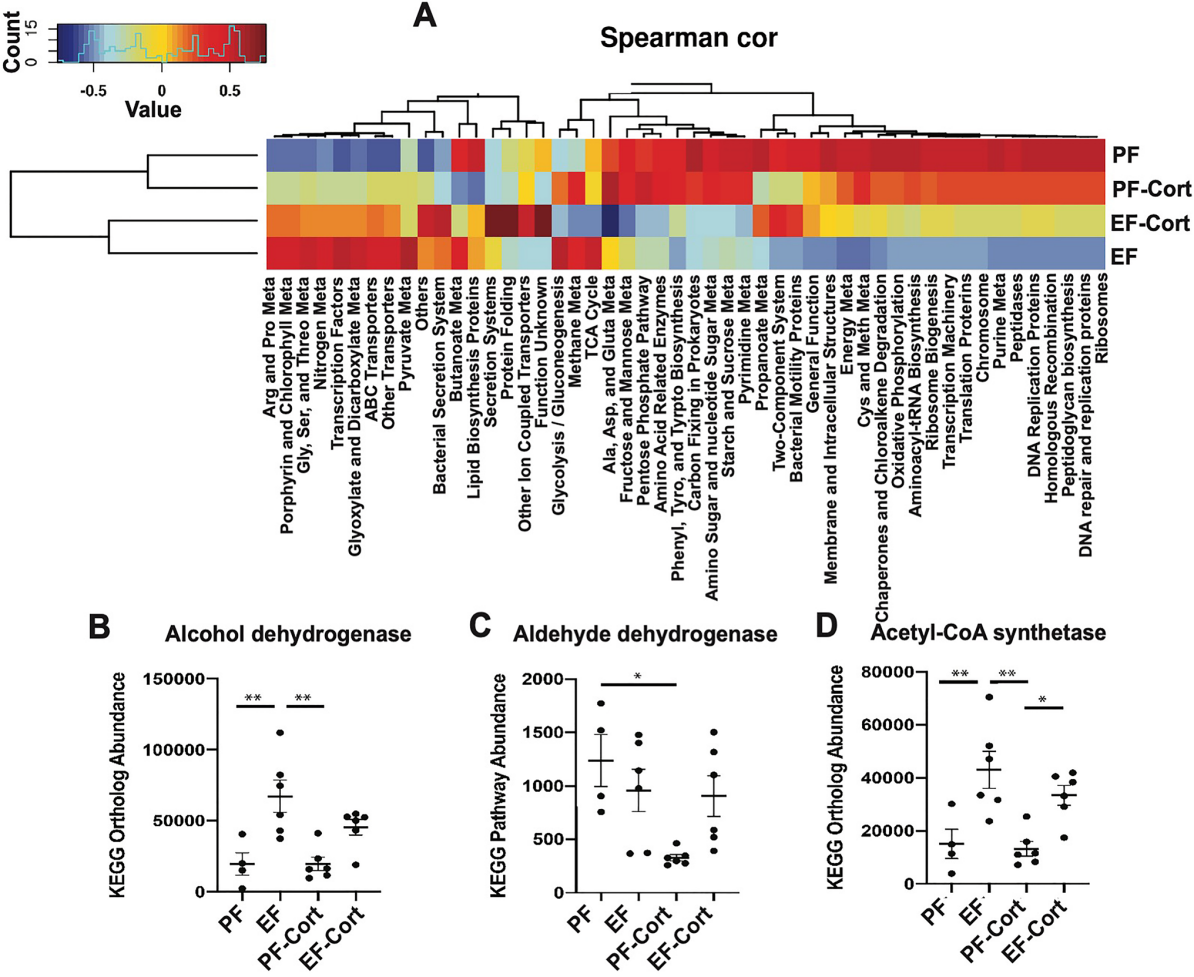
Corticosterone effect alcohol-induced intestinal dysbiosis: a functional analysis. Adult mice were fed a liquid diet with (EF) or without (PF) ethanol for four weeks. In some groups, animals were injected with corticosterone (CORT) daily. Animals in other groups were injected with the vehicle. Analysis of data from 16S rRNA-sequencing of fecal samples from different groups of mice is presented. (**A**) Functional categories associated with taxonomic composition were analyzed from the phylogenetic investigation of bacterial communities by PICRUSt. Spearman’s correlation on the most abundant microbial metabolic pathways in different groups is presented. (**B**–**D**) Effects of alcohol and corticosterone on the abundance of microbial categories expressing ethanol metabolizing enzymes, alcohol dehydrogenase (**B**), aldehyde dehydrogenase (**C**), and acetyl CoA synthetase (**D**).

To model the potential changes in microbiota community function, we applied Phylogenetic Investigation of Communities by Reconstruction of Unbiased States (PICRUSt) to predict functional differences associated with taxonomic composition in each experimental group. Data displayed as a Spearman’s correlation heatmap indicated significant functional differences between groups (Fig. 8A). Ethanol feeding decreased metabolic pathways involved in amino acid, carbohydrate, and energy metabolism, including oxidative phosphorylation, whereas it increased pathways related to ABC transporters and transcription factors. The combination of corticosterone with alcohol elevated the pathways involved in bacterial secretory systems, protein folding, and ion-coupled transporters. Combination of ethanol and corticosterone induced a robust down-regulation of butyrate-producing bacteria, associated with a dramatic reduction of TCA cycle activity. Data also show that ethanol increased the expression of alcohol metabolic enzymes, such as alcohol dehydrogenase (Fig. 8B), aldehyde dehydrogenase (Fig. 8C), and acetyl-CoA synthetase (Fig. 8D). Corticosterone had no significant effect on ethanol-induced upregulation of the ethanol-metabolic pathway.

**Figure 9:**
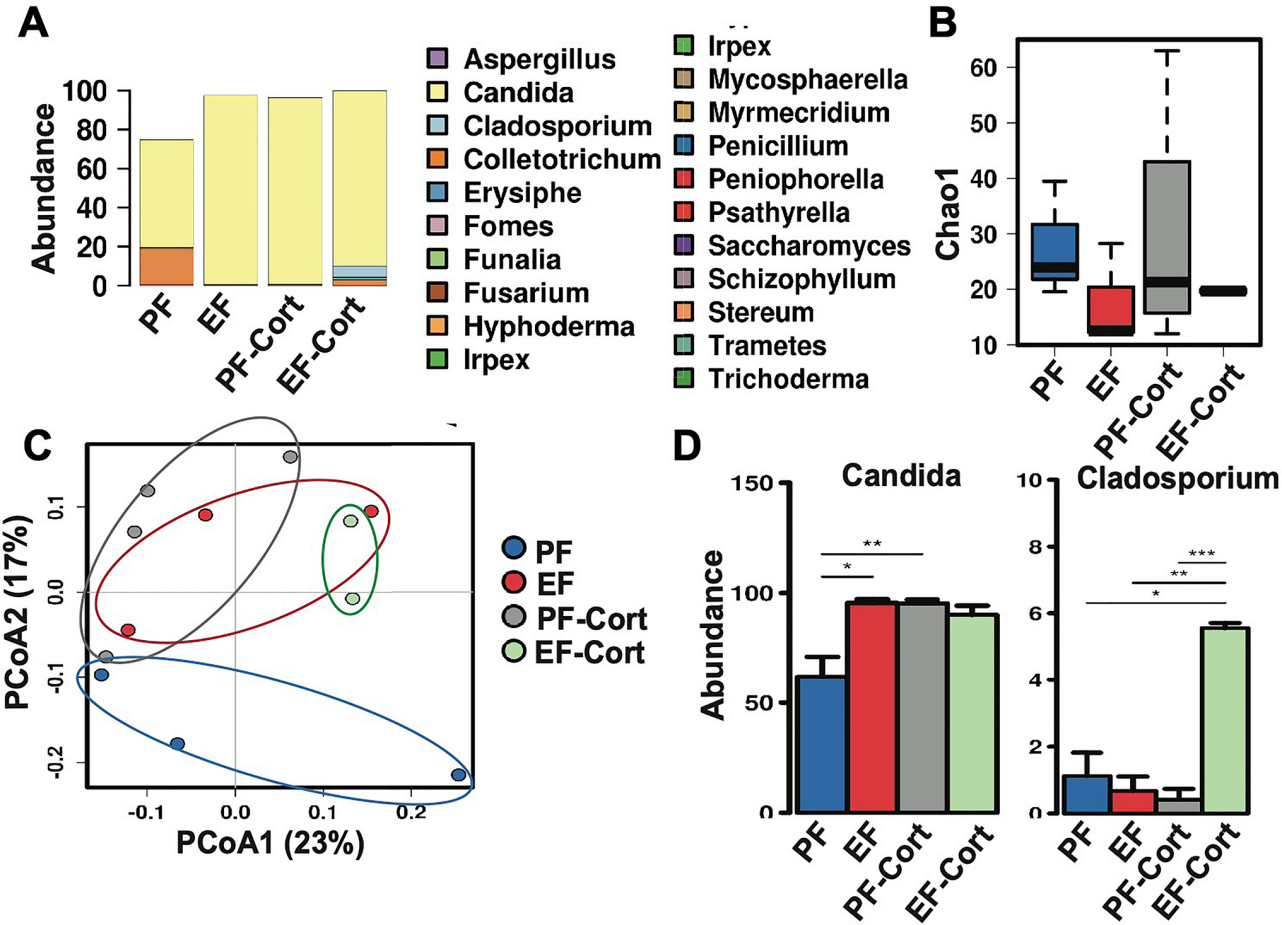
Corticosterone effect alcohol-induced dysbiosis of intestinal mycobiome. Adult mice were fed a liquid diet with (EF) or without (PF) ethanol for four weeks. In some groups, animals were injected with corticosterone (CORT) daily. Animals in other groups were injected with the vehicle. Analysis of data from 16S rRNA-sequencing of fecal samples from different groups of mice is presented. (**A**) Fungal phylum is displayed in each group. (**B**) Fungal α-diversity calculated by Chao1. (**C**) Principal coordinate analysis was used to visualize β-diversity in fungal populations with Jaccard dissimilarity. (**D**) Significantly altered fungal genus are displayed by ANOVA. *P < 0.05; **P < 0.01; ***P < 0.005.

Our metagenomic pipeline included Internal Transcribed Spacers (ITS) sequencing for fungal microorganisms collected from stool samples. In general, *Candida spp* dominated the mycobiota in this group of animals (Fig. 9A)*.* Despite the limited α-diversity found in the control group as assessed by the Chao1 (Fig. 9B) and Spearman correlation, ethanol feeding decreased fungal diversity further, driven largely by elevations in the relative abundance of *Candida spp*. Jaccard analysis indicated that β-diversity differed among the different groups (Fig. 9C). The abundance of *Candida spp* was similarly elevated by ethanol, corticosterone, and EtOH-CORT (Fig. 9D). Interestingly, the abundance of *Cladosporium*, a mold, was several-fold higher in the EtOH + CORT group than in the other groups.

## Discussion

A significant body of evidence indicates that the pathogenesis of ARD involves persistent inflammation and tissue injury by the synergistic actions of alcohol use and a second hit or multiple hits. Gut barrier dysfunction, dysbiosis of gut microbiota, endotoxemia, and systemic inflammation, are key foundational events involved in the pathogenesis of ARD, such as liver disease, pancreatitis, lung injury, and brain damage. A second insult may target any of these events to exacerbate alcohol-induced tissue injury. In the current study, we present evidence of the potential role of chronic stress and the stress hormone corticosterone in aggravating alcohol-induced gut barrier dysfunction, dysbiosis, endotoxemia, and systemic inflammation. The study also provides evidence of an essential role of corticosterone in enhancing the alcohol-induced tissue injury at the Gut-Liver-Brain axis.

Increased gut mucosal permeability due to chronic stress^44–47^ and alcohol consumption^14^ has been well documented. Our current study demonstrates that the combination of chronic stress and alcohol consumption synergistically elevates intestinal mucosal permeability by severe disruption of the intestinal epithelial TJ and AJ. Corticosterone is a primary stress hormone that plays an essential role in stress responses. The chronic elevation of corticosterone is associated with severe health problems^48,49^. The current study indicates that corticosterone, similar to chronic restraint stress, exacerbates alcohol-induced disruption of epithelial junctions and mucosal permeability. This finding suggests that corticosterone is a primary causal factor in the stress-induced promotion of alcohol-associated gut permeability.

The disruption of epithelial barrier function and LPS flux into mucosa leads to mucosal inflammatory responses. The potentiating effects of corticosterone on the ethanol-induced elevation of mRNA for *IL-1β, TNFα*, and *MCP1* suggest that corticosterone exacerbates alcohol-induced inflammatory responses in the intestinal mucosa. The mRNA for anti-inflammatory cytokine *IL-10* was reduced by ethanol feeding, which was further potentiated by corticosterone. Therefore, the upregulation of pro-inflammatory cytokines and downregulation of anti-inflammatory cytokines contribute to ethanol and corticosterone-induced inflammatory responses in the intestinal mucosa. The synergizing effects on barrier dysfunction and LPS absorption are likely responsible for the corticosterone-mediated potentiation of ethanol-induced inflammatory responses. However, we cannot rule out the direct impact of corticosterone on immune and epithelial cells. Glucocorticoids are conventionally anti-inflammatory and known for their application as therapeutics to treat many inflammatory diseases^50^. Our current study shows that corticosterone by itself does reduce the production of pro-inflammatory cytokines and chemokines. However, in the presence of alcohol, it increases the expression of pro-inflammatory cytokines and chemokines.

The alcohol and corticosterone-induced gut barrier dysfunction and intestinal mucosal inflammation were associated with endotoxemia. The elevation of plasma LPS by the combination of alcohol and corticosterone was higher than the sum of the individual effects of alcohol and corticosterone. Alcohol and corticosterone-induced endotoxemia were accompanied by a synergistic elevation of plasma TNFα, IL-1β, IL-6, and MCP1. These findings indicate that corticosterone potentiates alcohol-induced endotoxemia and systemic inflammation, although this hormone is known to be anti-inflammatory effects under certain conditions. Once again, these results indicate that corticosterone by itself suppress pro-inflammatory cytokine expression, but it increases these cytokines in the presence of alcohol^50^.

Two significant mechanisms define the cause of endotoxemia under various pathophysiologic conditions. First, disruption of intestinal epithelial TJ and barrier dysfunction allows diffusion of bacterial toxins from the colonic lumen into the portal circulation. In our in vivo studies, it is unclear whether corticosterone directly targets the intestinal epithelium to promote alcohol-induced gut permeability or indirect mechanisms mediate the response. A recent study demonstrated that ethanol sensitizes Caco-2 cell monolayers to acetaldehyde-induced TJ disruption and barrier dysfunction^16^. We used this model to determine whether corticosterone directly impact the intestinal epithelium and modulates alcohol-induced epithelial barrier dysfunction. Ethanol and acetaldehyde-induced TJ disruption and permeability in cell monolayers pre-treated with corticosterone, but not in sham-treated cell monolayers, indicated that corticosterone directly sensitizes intestinal epithelium for alcohol-induced epithelial barrier dysfunction.

The second mechanism contributing to endotoxemia and systemic inflammation is intestinal dysbiosis. A significant body of evidence indicates that chronic alcohol use results in intestinal dysbiosis, which contributes to alcohol-induced inflammation and organ damage substantially. Analysis of microbial abundance at the phyla level indicated that alcohol caused intestinal dysbiosis, and corticosterone modulated alcohol-induced dysbiosis. Gut microbial diversity was dramatically reduced by ethanol feeding. Corticosterone treatment partially blocked this effect of ethanol, indicating that corticosterone preserves bacterial diversity in alcohol-fed mice. Although the pair-fed control, ethanol, and ethanol with corticosterone groups shared somewhat similar α-diversity, the distinct β-diversity among these groups indicated substantial variation in microbial populations. This observation was confirmed by taxonomic analysis using Spearman’s correlation, LEfSe scoring, and network visualization. The modulation of bacterial populations by corticosterone in ethanol-fed mice included many genera of bacteria such as *Pseudomonas*, *Enterobacteriaceae*, *Brevundimonas*, *Prevotella,* and *Lactobacillus*. Significant functional differences are associated with the taxonomic composition of different groups of mice. Corticosterone upregulated the pathways related to protein folding, bacterial motility, and secretory system. We speculate that corticosterone increases the abundance of pathogenic bacteria, facilitates their invasive properties, and decreases the abundance of probiotics. Furthermore, a striking finding is that ethanol and corticosterone combined resulted in a robust reduction of butyrate-producing bacteria. This effect of ethanol and corticosterone is associated with a dramatic reduction of TCA cycle activity, the metabolic pathway necessary for butyrate production. Butyrate is known to have a mucosal protective role in the gut, and the butyrate prodrug, tributyrin, has been shown to prevent alcohol-induced tissue injury in mice^51,52^.

The liver is the first target of portal LPS. It is believed that gut barrier dysfunction leading to LPS absorption into the portal circulation is one of the primary factors involved in the pathogenesis of the alcohol-associated liver disease. The liver histopathology analyses, plasma AST activity, liver triglycerides, and cytokine/chemokine expression indicate that CRS and corticosterone aggravate ethanol-induced liver damage. This observation suggests that chronic stress and corticosterone promote the pathogenesis of alcohol-associated steatohepatitis. Although corticosterone by itself increased liver triglyceride, it reduced the expression of pro-inflammatory cytokines in the liver. However, the current data indicate that corticosterone is pro-inflammatory in the liver in the presence of alcohol. Although endotoxemia is a primary factor in systemic tissue injury, the other microbiome-based factors are likely involved in the mechanism of alcohol-associated tissue injury.

Neuroinflammation is one of the hallmark effects of chronic alcohol use^53^. The inflammatory responses in different brain regions are implicated in multiple phenotypes of AUD, such as addiction, anxiety, memory, and stress response^54^. The role of gut barrier dysfunction and resulting endotoxemia in alcohol-induced neuroinflammation is poorly understood. Our current study results indicate that chronic ethanol consumption and corticosterone treatment cause mild inflammation in the hypothalamus. However, a combination of ethanol and corticosterone caused a robust increase in the hypothalamic expression of pro-inflammatory cytokines and chemokines. In the normal stress response, corticosterone is released in response to stimulation of the hypothalamus. Corticosterone, in turn, suppresses hypothalamic corticosteroid release by negative feedback inhibition^55^. The current study indicates that alcohol consumption evokes a robust inflammatory response by the hypothalamus in the presence of corticosterone. The elevation of inflammatory cytokine expression in the hypothalamus by ethanol and corticosterone was associated with reduced *GR*, *BDNF*, and *TrkB* expression; however, corticosterone had no significant influence on these ethanol-induced effects. On the other hand, *CRHR1* receptor expression in the hypothalamus was elevated by ethanol and corticosterone with a robust synergy.

GR expression is high in the paraventricular nucleus (PVN) of the hypothalamus^56,57^. In a normal stress response, stress-induced increase in corticosterone production is terminated eventually by corticosterone-mediated feedback inhibition of PVN and CRH release^58,59^. A decrease in GR expression by chronic ethanol feeding indicates that the corticosterone-mediated feedback inhibition of the HPA axis is compromised, resulting in sustained elevation of plasma corticosterone. BDNF is expressed in the PVN of hypothalamus and plays an important role in the maintenance of various functions of HPA axis^60,61^, neuroprotection^62^, and synaptic plasticity^63^. Reduced expression of BDNF or TrkB decreased CRH expression and normal HPA functions^64^. Hence, ethanol-induced suppression of BDNF and TrkB in the hypothalamus may contribute to altered HPA axis and elevated plasma corticosterone. Further studies are needed to understand the interactions between the dysregulated HPA axis and alcohol-associated tissue injury.

The enhancing effects of corticosterone on alcohol effects raise the question of steroid-based therapy in alcohol-associated diseases. Prednisolone is a primary treatment for severe alcohol-associated hepatitis^65^; however, it offers only a short-term solution. Prednisolone is not useful for intermediate or long-term survival^66^. The primary mechanism of prednisolone therapy is its anti-inflammatory activity. Data from this study demonstrated that glucocorticoids elevated by alcohol use or administered exogenously caused gut barrier dysfunction, endotoxemia, systemic inflammation, and multiple organ damage. Therefore, it is not surprising that prednisolone is not an effective treatment strategy for the long-term survival of alcohol-associated hepatitis patients.

In summary, our current findings provide evidence of the role of chronic stress and the stress hormone corticosterone in potentiating alcohol-induced pathophysiology in the Gut-Liver-Brain axis. Corticosterone-induced sensitization of the intestinal epithelium for alcohol-induced TJ disruption and gut microbiota modulation are likely mechanisms underlying the stress-induced promotion of alcohol-associated tissue injury.

## Materials and methods

### Chemicals

Maltose dextrin was purchased from Bioserv (Flemington, NJ, USA). Regular Lieber-DeCarli EtOH diet was purchased from Dyets Inc (Cat# 710260; Bethlehem, PA, USA). Corticosterone, Horseradish peroxidase-conjugated anti-mouse IgG and anti-rabbit IgG antibodies, and anti-actin antibodies were purchased from Sigma-Aldrich (St Louis, MO, USA). Anti-ZO-1 antibody, anti-occludin antibody, Hoechst 33342 dye, and Alexa Fluor 488-conjugated phalloidin were purchased from Thermo Fisher Scientific (Waltham, MA, USA). Anti-E-cadherin and anti-β-catenin antibodies were purchased from BD Biosciences (San Jose, CA, USA). Alexa Fluor 488-conjugated anti-mouse IgG and Cy3-conjugated anti-rabbit IgG were purchased from Molecular Probes (Eugene, OR, USA).

### Animals and diets

Female C57BL/6 mice (8–10 weeks old; Harlan Laboratories, Houston, TX, USA) were used for chronic restraint stress and corticosterone treatment studies. All animal experiments were performed according to protocols approved by the UTHSC Institutional Animal Care and Use Committee. Animals were housed in an institutional animal care facility with 12:12 h light–dark cycles. Animals in all groups had free access to standard laboratory chow and water until the experiments. During the experiments, mice were fed the Lieber-DeCarli liquid diet as described before^67^.

### Stress and ethanol feeding

Animals were fed increasing doses of ethanol (EF) for 4 weeks (0% 2d, 1% 2 days, 2% 2 days, 4% 1 week, 5% 1 week, and 6% 1 week) in Lieber-DeCarli liquid diet as described before^67,68^. Control mice were pair-fed (PF) iso-caloric maltodextrin. In some groups, PF and EF mice were combined with one of the two types of stressors.

(1) *Chronic stress *Mice were subjected to chronic restraint stress (CRS) for 2 h daily during ethanol feeding. Non-stressed groups were maintained without food and water for 2 h daily (SHAM). For CRS, mice were physically restrained using the TV150 restrainer (Braintree Scientific, Braintree, MA) for 2 h daily (9–11 AM). Control mice were kept in cages with no food or water for 2 h. Five days after beginning CRS, animals are switched to liquid diet, but the CRS was continued until the day before euthanesia.

(2) *Corticosterone treatment *PF and EF mice were injected daily with corticosterone (25 mg/kg/day, s.c. for 4 weeks; CORT). Control groups were injected with the vehicle (VEH). Overall, 3 different studies were performed: (1) four wild-type mice—PF + SHAM, PF + CRS, EF + SHAM, and EF + CRS. (2) Four groups of wild-type mice—PF + VEH, PF + CORT, EF + VEH, and EF + CORT. In all experiments, animals were maintained in pairs to facilitate body temperature maintenance and prevent social isolation (an additional stressor).

### Gut permeability in vivo

Colonic and ileal mucosal permeability was evaluated as described previously^69^. At the end of each experiment, mice were intravenously injected with FITC-inulin (100 mg/ml solution; 2 μl/g body weight) via tail vein, and the fluorescence in plasma and intestinal luminal flushing was measured after one hour. Immediately following euthanasia, the ileum and colon were excised and flushed with 3 ml 09% saline. Flushing was briefly homogenized to break the solid contents and centrifuged at 2000×*g* to collect the supernatant and measure fluorescence in a plate reader (BioTek, Winooski, VT). Fluorescence values in the luminal flushing were normalized to fluorescence values in corresponding plasma samples and calculated as the inulin load percentage.

### Immunofluorescence microscopy

Immunofluorescence staining was performed as described previously^69^. Cryosections (10 μm) were fixed in acetone: methanol (1:1), permeabilized with 0.2% Triton X-100 in PBS for 10 min and blocked in 4% nonfat milk in Triton-Tris buffer. Tissue sections were incubated with primary antibodies (mouse monoclonal anti-occludin and rabbit polyclonal anti–ZO-1 antibodies or mouse monoclonal E-cadherin and rabbit polyclonal anti-β-catenin antibodies) for one hour. It was then incubated for another hour with the secondary antibodies (Alexa Fluor 488-conjugated anti-mouse IgG and Cy3-conjugated anti-rabbit IgG antibodies). Hoechst 33342 dye was added during the last 10 min of incubation. The actin cytoskeleton was stained with Alexa Fluor 488-conjugated phalloidin. The fluorescence was examined using a Zeiss 710 confocal microscope (Carl Zeiss GmbH, Jena, Germany) and processed as previously described^68,69^. All images for tissue samples from different groups were collected and processed under identical conditions.

### RT-PCR

Tissues (ileum, distal colon, liver, and brain regions) were dissected immediately after decapitation and stored at − 80 °C in RNAlater (Ambion, Austin, TX, USA). Individual tissues were homogenized in TRIzol (Invitrogen, Carlsbad, CA, USA), and RNA was isolated. For the hypothalamus, the brain was removed and placed ventral side up. The curved part of the forceps was pushed down around the hypothalamus was pushed down using curved forceps and gently spooned by pinching it out. Immediately, the tissue was placed in 2 ml tubes with 0.5 ml of RNAlater.

RT-qPCR was performed as described before^69^. Total RNA (1.5 μg) was used to generate cDNAs using the ThermoScript RT-PCR System for first-strand synthesis (Thermo Fisher Scientific). Quantitative PCR (qPCR) reactions were performed using cDNA mix (cDNA corresponding to 35 ng RNA) with 300 nM of primers in a final volume of 25 μl of 2 times concentrated RT2 Real-Time SYBR Green/Rox Master Mix (Qiagen, Germantown, MD, USA) in an Applied Biosystems QuatStudio 6-Flex Real-Time PCR instrument (Thermo Fisher Scientific). The cycle parameters were as follows: 50 °C for 2 min, one denaturation step at 95 °C for 10 min, and 40 cycles of denaturation at 95 °C for 10 s followed by annealing and elongation at 60 °C. The relative gene expression of each transcript was normalized to GAPDH using the ΔΔCt method. Sequences of primers used for qPCR are provided in Table S1.

### Microbiome analyses

Microbiome analyses of fecal samples were performed as described recently^70^.

#### DNA extraction and Illumina MiSeq sequencing

Stool samples were resuspended in 500 μL of TNES buffer containing 200 units of lyticase and 100 μL of 0.1/0.5 (50/50 Vol.) zirconia beads. Incubation was performed for 20 min at 37 °C. Following mechanical disruption using ultra-high-speed bead beating, 20 μg of proteinase K was added to all samples, and they were incubated overnight at 55 °C with agitation. Total DNA was extracted using chloroform-isoamyl-alcohol mixture (25:24:1), and total DNA concentration per mg stool was determined by qRT-PCR. Purified DNA samples were sent to the Argonne National Laboratory (Lemont, IL) for amplicon sequencing using the NextGen Illumina MiSeq platform. Blank samples passed through the entire collection, extraction, and amplification process remained free of DNA amplification.

#### Bioinformatics

Sequencing data were processed and analyzed using QIIME (Quantitative Insights into Microbial Ecology) 1.9.1 Sequences were first demultiplexed, then denoised, and clustered into sequence variants. For bacteria, we rarified to a depth of 10,000 sequences. Representative bacterial sequences were aligned via PyNAST, with taxonomy assigned using the RDP Classifier. Processed data were then imported into Calypso 8.84 for further analysis and data visualization^71^. The Shannon index was used to quantify α-diversity^72,73^. Bray–Curtis analysis was used to quantify β-diversity, and the differences were compared using PERMANOVA with 999 permutations. For fungi, sequences were aligned, and taxonomy was assigned using the UNITE metabarcoding (dynamic setting) database^74^. Fungal OTUs were rarified at a depth of 300 sequences for α-diversity using Chao1 and β-diversity based on Jaccard dissimilarities at the OTU level^75^. As with bacteria, beta diversity was then assessed using permutational multivariate analysis of variance (PERMANOVA). To quantify the relative abundance of taxa between groups^76^, we utilized ANOVA adjusted using the Bonferroni correction and FDR for multiple comparisons. We used linear discriminant analysis of effect size (LEfSe) to test for significance and perform high-dimensional biomarker identification^77^. Network analysis was generated from Spearman’s correlations. Positive correlations with a false discovery rate (FDR)-adjusted, *p* < 0.05 were presented as an edge. PICRUSt was performed by picking closed OTU tables against GreenGenes, as previously described (https://www.nature.com/articles/nbt.2676). Following pathway prediction, a heat map was generated for the 50 most abundant pathways using Spearman’s correlations. Predictive metagenomics was performed using Phylogenetic Investigation of Communities by Reconstruction of Unobserved States (PICRUSt), where abundances of metabolic pathways were then calculated by Spearman’s rank correlation coefficient and presented in a heatmap or specific alcohol-related enzymes were displayed as ANOVA. (See Citation in comments).

### Plasma endotoxin assay

Plasma endotoxin concentrations were measured using the Pierce LAL Chromogenic Endotoxin Quantitation Kit (Cat# 88282; Thermo Fisher Scientific).

### Liver histopathology

Liver and colon tissues were fixed in 10% buffered formalin, and 8 µm thick paraffin-embedded sections were stained with hematoxylin and eosin (H&E) as described recently^69^. For steatosis analysis, frozen liver sections were fixed in 4% paraformaldehyde, stained with Oil Red O (Sigma-Aldrich), and rinsed with 60% isopropanol; nuclei were stained with hematoxylin. Stained sections were imaged by a Nikon 80Ti Microscope (Nikon, Tokyo, Japan) using a 40× objective lens and a color camera.

### Plasma transaminase assay

Plasma ALT and AST activities were measured by colorimetric assay using specific kits from Cayman Chemicals (Ann Arbor, MI, USA).

### Triglyceride assay

Liver lipids were extracted by digestion with 3 M potassium hydroxide (in 65% ethanol) at room temperature for 24 h. Liver triglycerides were quantified by the glycerol phosphate oxidase method using an assay kit from Beckman Coulter (Brea, CA, USA).

### Cell culture

Caco-2_bbe2_ cells (American Type Culture Collection, Manassas, VA, USA, Manassas, VA, USA) were grown under standard cell culture conditions as previously described^44^. Experiments were conducted using cells grown in Transwell inserts (6.5 mm; Corning, Corning, NY, USA) for ten days.

### Cell treatments

Cell monolayers were pre-treated with corticosterone (0, 0.5, 1.0, or 10.0 μM) for 24 h, followed by incubation with 100 μM acetaldehyde and 20 mM ethanol for 1–3 h.

### Epithelial barrier function

Barrier function was evaluated by measuring transepithelial electrical resistance (TER) and inulin permeability as described before^69^. TER was measured using a Millicell-ERS Electrical Resistance System (MilliporeSigma), and macromolecular permeability was evaluated by measuring the unidirectional flux of FITC-inulin. The basal TER values for Caco-2 cell monolayers were 400–500 Ω/cm^2^.

### Statistical analyses

The differences among multiple groups were first analyzed by ANOVA via GraphPad Prism 6.0 software (GraphPad Software, La Jolla, CA, USA) as described before^69^. When statistical significance was detected, Tukey's t-test was used to determine the significance between multiple testing groups and the corresponding controls. Statistical significance was established at 95%.

### Microbiota data access

Data were deposited in the NCBI Sequence Read Archive (SRA) at: https://submit.ncbi.nlm.nih.gov/subs/sra/SUB7619938/overview.

## Supplementary Information

Supplementary Information

## Abbreviations

AA: Acetaldehyde
AJ: Adherens junction
ALT: Alanine aminotransferase
AST: Aspartate aminotransferase
AUD: Alcohol use disorder
BDNF: Brain-derived neurotrophic factor
CRHR1: Corticotropin-releasing hormone receptor-1
CRS: Chronic restraint stress
CORT: Corticosterone
EtOH: Ethanol
GLB: Gut–liver–brain axis
GR: Glucocorticoid receptor
HPA: Hypothalamic–pituitary–adrenal
LPS: Lipopolysaccharide
TJ: Tight junction
TLR4: Toll-like receptor 4
TNF: Tumor necrosis factor
TrkB: Tropomyosin receptor kinase B
ZO-1: Zonula occludens
