# Supplementary material for: Chronic stress and corticosterone exacerbate alcohol-induced tissue injury in the gut-liver-brain axis
Source: Sci Rep. 2021 Jan 12;11:826. doi: 10.1038/s41598-020-80637-y (PMC7804442; doi:10.1038/s41598-020-80637-y)
Supplement: Supplementary file 1 — Supplementary Information [file 41598_2020_80637_MOESM1_ESM.pdf]

SUPPLENTAL INFORMATION

Chronic Stress and Corticosterone Exacerbate Alcohol-Induced Tissue Injury at the Gut-Liver-Brain Axis

[1*]Pradeep K. Shukla, [1*]Avtar S. Meena, [1]Kesha Dalal, [1]Cherrie Canelas, [1]Geetha Samak, [2]Joseph F. Pierre, [1¶]RadhaKrishna Rao

Departments of [1]Physiology and [2]Pediatrics, College of Medicine, University of Tennessee Health Science Center, Memphis, TN, USA

[¶]***Address for correspondence:***
R. K. Rao, Ph.D., AGAF
Department of Physiology
University of Tennessee Health Science Center
3 N Dunlap, Suite S303
Memphis, TN 38103
Phone: (901) 448-3235
Fax: (901) 448-3139
Email: rrao2@uthsc.edu

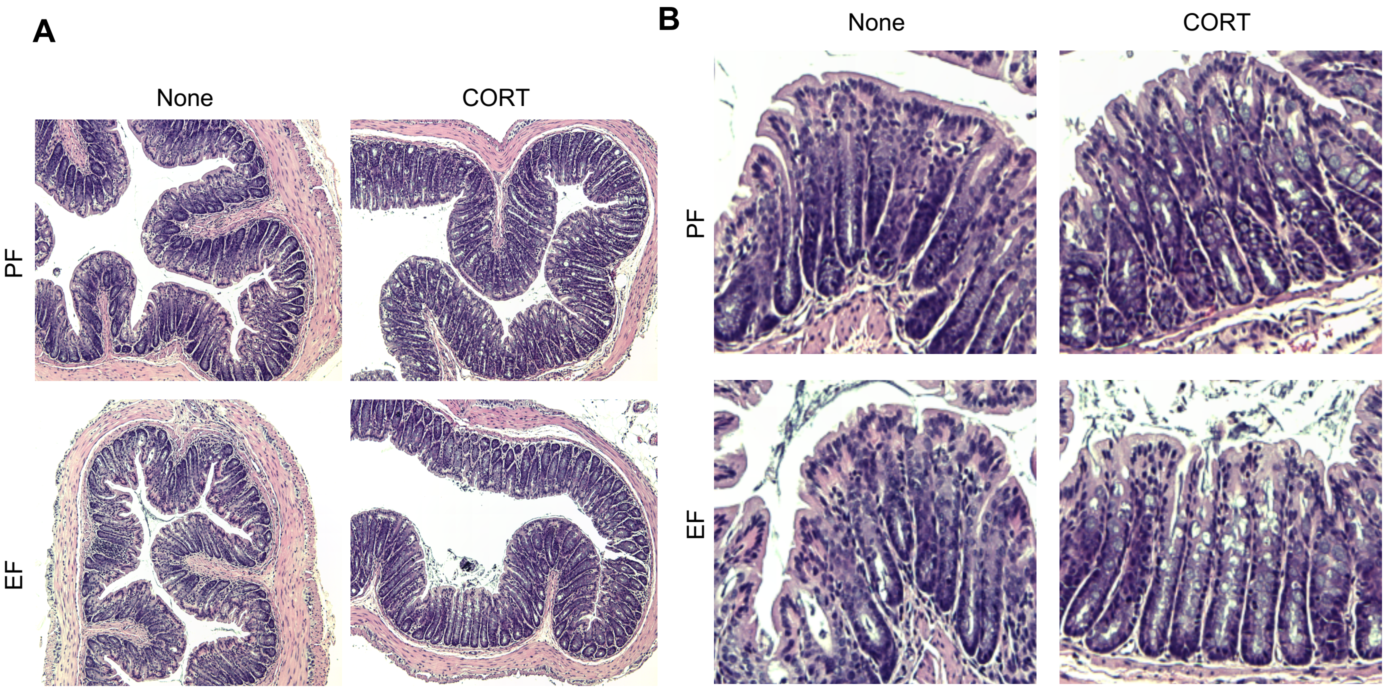

Figure S1
Effects of alcohol and corticosterone on colon.
Adult mice were fed a liquid diet with (EF) or without (PF) ethanol for four weeks. In some groups, animals were injected with corticosterone (CORT) or vehicle daily. Formaldehyde fixed colonic sections were stained with H & E and imaged by light microscopy at 10x (A) or 40x (B) magnification.

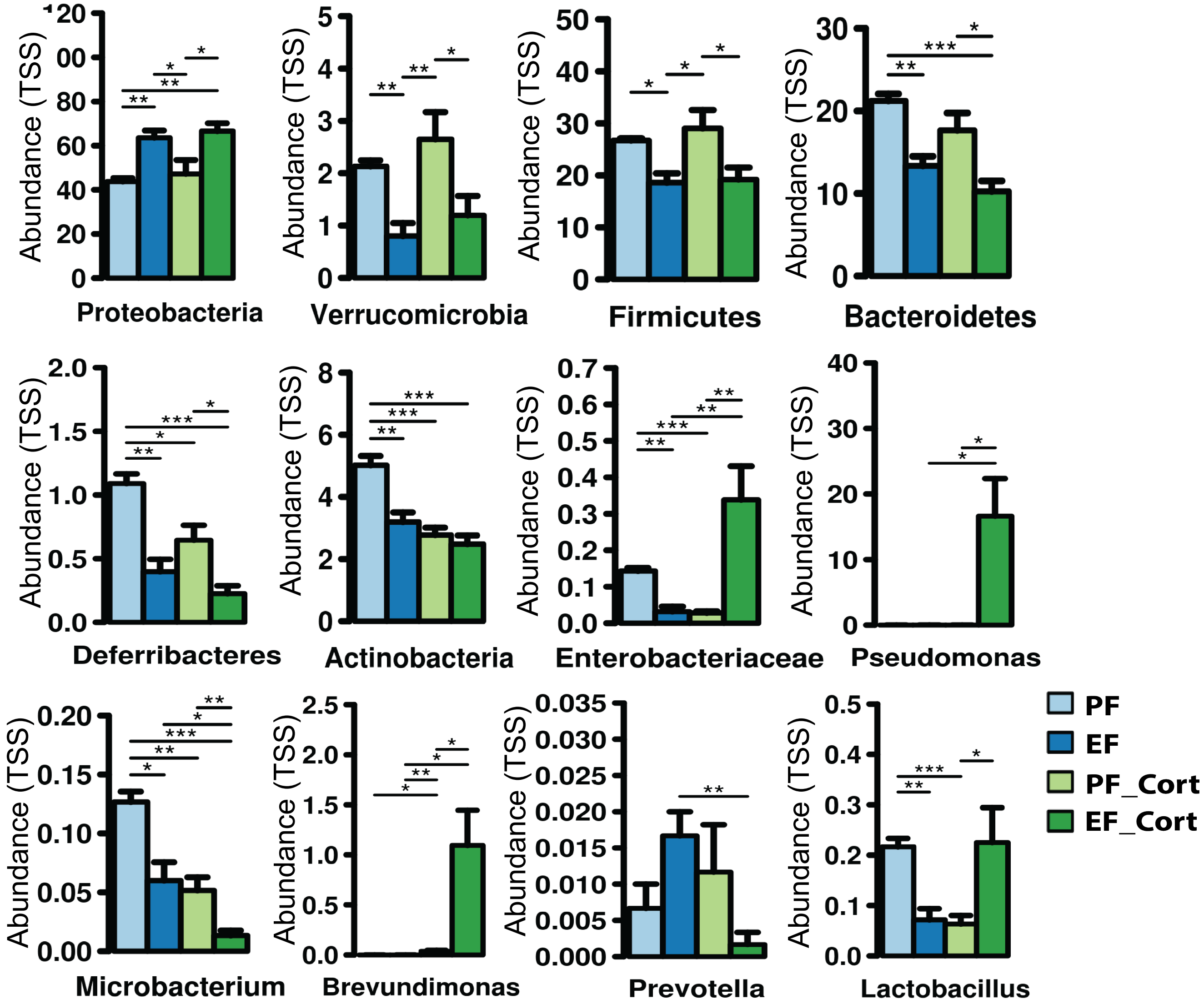

Figure S2: The effects of alcohol and corticosterone on alcohol-induced colonic dysbiosis.

Adult mice were fed a liquid diet with (EF) or without (PF) ethanol for four weeks. In some groups, animals were injected with corticosterone (Cort) daily. Animals in other groups were injected with the carrier. Analysis of data from 16S rRNA-sequencing of fecal samples from different groups of mice is presented. **A**: relative abundance of different phyla of bacteria. Asterisks indicate significant differences between groups. * $P < 0.05$; ** $P < 0.01$; *** $P < 0.005$.

Table S1: PCR primer sequences

Gene	5'-3' Sequence	
**IL-1β**	Forward: GCAACTGTTCCTGAACTCAACT	
	Reverse: ATCTTTTGGGGTCCGTCAACT	
**IL-6**	Forward: TAGTCCTTCCTACCCCAATTTCC	
	Reverse: TTGGTCCTTAGCCACTCCTTC	
**TNF-α**	Forward: CCCTCACACTCAGATCATCTTCT	
	Reverse: GCTACGACGTGGGCTACAG	
**IL10**	Forward: GCTCTTACTGACTGGCATGAG	
	Reverse: CGCAGCTCTAGGAGCATGTG	
**TLR4**	Forward: ATGGCATGGCTTACACCACC	
	Reverse: GAGGCCAATTTTGTCTCCACA	
**MyD88**	Forward: TCATGTTCTCCATACCCTTGGT	
	Reverse: AAACTGCGAGTGGGGTCAG	
**MCP-1/CCL2**	Forward: TTAAAAACCTGGATCGGAACCAA	
	Reverse: GCATTAGCTTCAGATTTACGGGT	
**CCL5/RANTES**	Forward: GCTGCTTTGCCTACCTCTCC	
	Reverse: TCGAGTGACAAACACGACTGC	
**TrkB**	Forward: CTGGGGCTTATGCCTGCTG	
	Reverse: AGGCTCAGTACACCAAATCCTA	
**BDNF**	Forward: TCATACTTCGGTTGCATGAAGG	
	Reverse: AGACCTCTCGAACCTGCCC	
**GR**	Forward: AGCTCCCCCTGGTAGAGAC	
	Reverse: GGTGAAGACGCAGAAACCTTG	
**Crhr1**	Forward: GTGGACCCCCTAACAACAGTG	
**GAPDH**	Reverse: CGGGAAGACACGCGATTATCA<br>Forward: CTGCACCACCAACTGCTTAG<br>Reverse: GGGCCATCCACAGTCTTCT	
